# Evaluation of the implant diameter on the initial-stability of narrow- and standard-diameter implants placed in simulated Type-I and Type-IV bone-blocks

**DOI:** 10.12669/pjms.37.3.3943

**Published:** 2021

**Authors:** Saad Alresayes, Sameer A Mokeem, Aasem M Alhenaki, Fahim Vohra, Tariq Abduljabbar

**Affiliations:** 1 Saad Alresayes, BDS Department of Prosthetic Dental Science, College of Dentistry, King Saud University, Riyadh 11545, Saudi Arabia; 2 Sameer A. Mokeem, BDS, MSc Department of Periodontics and Community Dentistry, King Saud University, Riyadh 11545, Saudi Arabia; 3 Aasem M Alhenaki, BDS, MSc Department of Prosthetic Dental Science, College of Dentistry, King Saud University, Riyadh 11545, Saudi Arabia; 4 Fahim Vohra, MRD, PhD Department of Prosthetic Dental Science, College of Dentistry and Research Chair for Biological Research in Dental Health, College of Dentistry, Riyadh 11545, Saudi Arabia; 5 Tariq Abduljabbar, BDS, DMSc Department of Prosthetic Dental Science, College of Dentistry and Research Chair for Biological Research in Dental Health, College of Dentistry, Riyadh 11545, Saudi Arabia

**Keywords:** Dental implant, In-Vitro, initial stability, Narrow diameter, Simulated bone, Standard diameter

## Abstract

**Objective:**

A comparison of the initial stability of narrow- and standard-diameter implants (SDIs) placed in Type-I and Type-IV bone-blocks is not yet reported. The aim was to evaluate in-vitro the influence of implant diameter on the initial stability of narrow- and standard-diameter implants (SDIs) placed in simulated Type-I and Type-IV bone-blocks.

**Methods:**

The present experimental in-vitro study was performed between July and September 2020 at the Specialist Dental Practice, Riyadh, Saudi Arabia. Narrow- and standard-diameter implants were placed 3-mm apart in simulated soft (Type-IV) and dense (Type-I) bone blocks by a trained and calibrated investigator. In groups A (Type-IV bone blocks) and B (Type-I bone blocks), implants were inserted using an insertion-torque and drilling-speed of 15-30 Ncm and 1000-1500 rpm, respectively with the implant collar at the crest of simulated bone blocks. In all samples, initial-stability was recorded using resonance frequency analysis (RFA). Sample-size estimation was done and group-comparisons were carried out. A P-value of 0.01 or less reflected statistical significance.

**Results:**

In Groups-A and -B, 44 (22 NDIs and 22 SDIs) and 44 (22 NDIs and 22 SDIs) were placed. In group-A, the mean RFA values for NDIs and SDIs were 68.5 ± 3.5 and 69.1 ± 2.4, respectively. In Group-B, the mean RFA values for NDIs and SDIs were 78.06 ± 9.6 and 75.3 ± 5.2. RFA values among NDIs and SDIs in groups A and B were similar.

**Conclusion:**

The NDIs and SDIs show comparable initial-stability when positioned in simulated Type-I and Type-IV bone blocks.

## INTRODUCTION

Mechanical inter-locking between the implant threads and surrounding alveolar bone is critical for osseointegration;^1^ and helps provide initial-stability that plays an integral role in stabilizing dental implants.^2^ Factors that affect initial-stability include implant surface morphology, surgical technique used (such as bone spreading and osseous condensation) and implant geometry.^3^ Another factor that influences initial-stability is the eminence of bone that contains the implant. Most of the classifications that have been reported in relation to bone quality rely on the relative ratios of spongy-trabecular to cortical-compact bone.^4^ In summary, Type-I bone is the densest and least vascular; whereas Type-II bone is a blend of cortical bone including marrow cavities. Type-III bone is trabecular in nature, and Type-IV has a thin cortex with less dense trabeculae.

The regular- or standard-diameter implants (SDIs) have a diameter of at least 4mm and bone expansion/augmentation protocols are often warranted prior to their placement particularly in the posterior maxilla.^5^ However, with the advent of narrow-diameter implants (NDIs), the need for complicated surgical procedures and related adverse events such as uncontrolled bleeding, wound dehiscence and nerve damage have markedly reduced in contrast to standard-diameter implants.^6^ An agreement regarding the absolute diameter of NDIs is yet to be reached; however, in indexed literature, dental implant with a diameter of up to 3.5 mm are considered NDIs.^7^,^8^ According to Ma M et al.^9^ NDIs can successfully support single-tooth supported implants in the posterior maxillae in a way similar to regular or SDIs. Moreover, 3-year prosthesis success rates for NDIs and SDIs have been reported as 89.25% and 96.55%, respectively.^9^ Nevertheless, there is a lack of literature regarding the comparison of initial-stability of NDIs and SDIs placed in less dense bones with a thin cortex, such as the posterior maxilla. It is theorized that there is no difference in the initial-stability of narrow- and SDIs placed in artificial Type-I and Type-IV bone-blocks. The purpose was to evaluate the influence of implant diameter on the initial-stability of NDIs and SDIs placed in simulated Type-I and Type-IV bone-blocks.

## METHODS

The present experimental in-vitro study was performed in artificial Type-IV bone-blocks. In this regard, a prior Institutional Ethical Review Board approval was not required. The study was performed between July and September 2020 at Specialist Dental Practice and research Center, Riyadh, Saudi Arabia.

### Implant-characteristics and placement:

In the present study, NDIs and SDIs were used. The lengths and diameters of NDIs and SDIs were 11 mm and 3.5 mm, and 4.1 and 14 mm, respectively. Geometrically, all implants were platform-switched with a tapered design. All implants had moderately rough surfaces. In the simulated type-I and type-IV bone-blocks, the NDIs and SDIs were perpendicularly placed 3-mm apart at a drilling-speed and insertion-torque of 1000-1500 rpm and 15-30 Ncm, respectively. The density of the simulated type-I (Group-A) and type-IV (Group-B) bone-blocks was 0.64 and 0.16 g/cm^3^, respectively. All implants were inserted until the implant collar reached the crest of simulated bone-blocks. All implants were placed by one investigator (*Kappa* score 0.94).

### Evaluation of initial stability:

In groups A and B, initial-stability was recorded by one investigator (*Kappa* score: 0.9) using RFA (Osstell, Integration Diagnostics Ltd., Goteborgsvagen, Sverige). Three readings were recorded for each implant with an interval of 30 seconds between each reading. The mean of the 3 readings was recorded as the corresponding RFA value.

### Statistical analysis:

The methodology was review by an independent statistician. A computer-based software (SPSS-20, Chicago, IL, USA) was used to perform the statistical analysis. The normality was measured by Shapiro-Wilk test. The RFA values were presented as means ± standard deviation; and group-comparisons were accomplished using the Student *t*-test. A P-value of 0.01 or less reflected statistical significance. Based upon polit results, a power-analysis was done. It was estimated that inclusion of 22 NDIs and 22 SDI in groups A and B would provide a power of 95% to the study with an alpha of 0.05.

## RESULTS

In Groups A and B, 44 (22 NDIs and 22 SDIs) and 44 (22 NDIs and 22 SDIs) were placed. In group-A, the mean RFA values for NDIs and SDIs were 68.5 ± 3.5 and 69.1 ± 2.4, respectively. In Group-B, the mean RFA values for NDIs and SDIs were 78.06 ± 9.6 and 75.3 ± 5.2. There was no significant variation in the RFA values among NDIs and SDIs in both groups.

## DISCUSSION

Various techniques such as Perio-test, reverse torque test, resonance-frequency-analysis (R-F-A), and assessment of implant-stability-quotient (ISQ) are used to assess implant stability^10^-^13^; however, a global agreement regarding the most suitable mode of assessment is yet to be reached. There is a dearth of studies that have compared initial-stability of NDIs with SDI in relation to density of host bone. To the authors’ knowledge, the present experimental study is the first one to compare the initial stability of NDIs and SDIs placed in simulated bone with varying densities. From a clinical perspective, the present experimental results indicate that bone density is not a contra-indication towards the achievement of primary or initial stability following implant placement. Results of the present in-vitro experiment are in accordance with the proposed hypothesis as the RFA values for NDIs and SDIs placed in Type-I and Type-IV bone-blocks showed no statistically significant difference. In other words, NDIs demonstrate initial-stability similar to SDIs when placed in bone-blocks of varying density (type-I and type-IV). One study reported that implants placed by well-trained operators show greater ISQ values than inexperienced or novice operators.^14^ Results of a literature review reported that there is an inversely proportional relationship between implant failure and operator skills.^15^ According to Romanos GE et al.^16^ experienced clinicians achieved higher ISQ values for implants placed in poor quality bone. Nevertheless, it has also been documented that operators’ experience is rather a secondary factor that affects the primary stability of implants.^17^ In the present study, all implants were placed by an experienced oral surgeon; and this factor may have contributed towards achievement of sufficient initial-stability of NDIs and SDIs in Type-I and Type-IV simulated bone-blocks.

In the present study, the NDIs and SDIs had a tapered design and had moderately rough surfaces. The authors applaud the results of a recent experimental study^14^ that investigated the influence of the implants’ apical portion on initial-stability. In this study^14^, the implants had a progressive thread design and were inserted in blocks that simulated dense (type-II) bone. Moreover, the NDIs used in the *in-vitro* study by Romanos GE et al.^14^ and the present investigation were the same that is, 11 mm and 3.5 mm in lengths and diameter, respectively. The authors concluded that implant-stability in the apical region provides to up 43% of the total stability to an implant.^14^ It is postulated that the threads condense the bone and alongside “inter-lock” implant threads firmly into surrounding bone throughout the length of the implant during and after insertion. It has also been reported that rough-surfaced implants demonstrate superior initial-stability than smooth-surfaced or machined implants;^18^ and from a clinical perspective, dental implants with moderately-rough surfaces have significantly higher success rates than implants with machined-surfaces.^18^ In our experiment, NDIs and SDIs had moderately-rough implant surfaces and this factor may have also contributed towards the achievement of sufficient initial-stability. With regards to mode of assessment of implant stability, a consensus regarding the most precise and accurate method is yet to be reached. However, assessment of RFA and ISQ are considered considerably if not equally reliable methods to gauge implant stability.^10^ In the present study, only RFA was done to assess implant-stability as recommended in previous in clinical implantology and associated research.^10^

### Limitations on the study:

A major limitation of the present study is that all implants were placed in simulated bone blocks. Achievement of primary stability is critical for the long-term success and survival of dental implants;^19^ however, it is challenging to replicate these results into a clinical setting as a variety of factors influence osseointegration and peri-implant soft-tissue status. Such factors encompass smoking, systemic illnesses including diabetes mellitus, and treatments such as irradiation and chemotherapy that jeopardize crestal bone should be taken into account in a clinical setting.^20^-^24^ It is hypothesized that in a clinical scenario, NDIs and SDIs demonstrate sufficient initial-stability and can remain mechanically stable when placed in the posterior maxilla, which comprises of type-IV bone. It is also speculated that NDIs and SDIs positively influence immediate functional loading protocols in low density bone. This requires additional studies preferably randomized controlled clinical trials that are well-designed and power-adjusted. Such factors may compromise implant stability and overall survival of NDIs and SDIs. In other words, the significance of treatment planning, patient selection and patient education remain crucial and this is independent of bone and implant characteristics.

## CONCLUSION

The NDIs and SDIs show comparable initial-stability when placed in simulated Type-I and Type-IV bone-blocks.

### Authors’ Contribution:

**TA and FV** conceived and designed the study and edited the manuscript; and are responsible and accountable for the accuracy or integrity of the work.

**FV** wrote the methods and did statistical analysis.

**SAM, SA & AMA** did data collection and manuscript writing.

**TA, AMA and FV** did review and final approval of manuscript.
